# Research on a UAV spray system combined with grid atomized droplets

**DOI:** 10.3389/fpls.2023.1286332

**Published:** 2024-01-03

**Authors:** Xiuyun Xue, Yu Tian, Zhenyu Yang, Zhen Li, Shilei Lyu, Shuran Song, Daozong Sun

**Affiliations:** ^1^ College of Electronic Engineering (College of Artificial Intelligence), South China Agricultural University, Guangzhou, China; ^2^ Division of Citrus Machinery, China Agriculture Research System of Ministry of Finance the People 's Republic of China and Ministry of Agriculture and Rural Affairs of the People 's Republic of China, Guangzhou, China; ^3^ Guangdong Provincial Agricultural Information Monitoring Engineering Technology Research Center, Guangzhou, China; ^4^ Pazhou Lab, Guangzhou, China

**Keywords:** grid atomization, agricultural unmanned aerial vehicle, droplet drift, deposition effect, machine learning prediction

## Abstract

**Backgrounds:**

UAVs for crop protection hold significant potential for application in mountainous orchard areas in China. However, certain issues pertaining to UAV spraying need to be addressed for further technological advancement, aimed at enhancing crop protection efficiency and reducing pesticide usage. These challenges include the potential for droplet drift, limited capacity for pesticide solution. Consequently, efforts are required to overcome these limitations and optimize UAV spraying technology.

**Methods:**

In order to balance high deposition and low drift in plant protection UAV spraying, this study proposes a plant protection UAV spraying method. In order to study the operational effects of this spraying method, this study conducted a UAV spray and grid impact test to investigate the effects of different operational parameters on droplet deposition and drift. Meanwhile, a spray model was constructed using machine learning techniques to predict the spraying effect of this method.

**Results and discussion:**

This study investigated the droplet deposition rate and downwind drift rate on three types of citrus trees: traditional densely planted trees, dwarf trees, and hedged trees, considering different particle sizes and UAV flight altitudes. Analyzing the effect of increasing the grid on droplet coverage and deposition density for different tree forms. The findings demonstrated a significantly improved droplet deposition rate on dwarf and hedged citrus trees compared to traditional densely planted trees and adopting a fixed-height grid increased droplet coverage and deposition density for both the densely planted and trellised citrus trees, but had the opposite effect on dwarfed citrus trees. When using the grid system. Among the factors examined, the height of the sampling point exhibited the greatest influence on the droplet deposition rate, whereas UAV flight height and droplet particle size had no significant impact. The distance in relation to wind direction had the most substantial effect on droplet drift rate. In terms of predicting droplet drift rate, the BP neural network performed inadequately with a coefficient of determination of 0.88. Conversely, REGRESS, ELM, and RBFNN yielded similar and notably superior results with a coefficient of determination greater than 0.95. Notably, ELM demonstrated the smallest root mean square error.

## 1 Introduction

Agricultural aviation crop protection has significant advantages such as low terrain restrictions, high spray efficiency, and the ability of downdrafts to promote droplet deposition on both sides of the leaves (He et al., 2017). However, the formulation used in the current unmanned aerial vehicle (UAV) crop protection spraying operation is typically based on ground-based machinery. Due to the limited payload capacity of UAVs, the application rate per unit area needs to be reduced to ensure a certain operational efficiency. To achieve the same operational effect, it is necessary to increase the concentration of the liquid and reduce the size of the droplets. However, high-concentration and small-droplet sprays are prone to problems such as pesticide evaporation and drift, resulting in pesticide waste and pollution (Hilz and Vermeer, 2013; Lou et al., 2018). Therefore, there is an urgent need to develop efficient crop spraying techniques for agricultural aviation to reduce secondary disasters and improve the utilization rate of agricultural pesticides(Hu et al., 2022).

To improve the deposition rate and reduce the drift rate of pesticide solutions on targets, researchers have explored the relationship between the number of rotors, flight speed, and altitude of unmanned aerial vehicles (UAVs) and the quality of droplet deposition and spraying effectiveness, aiming to further improve the efficiency of UAV plant protection spraying. Martinez-Guanter (2020) compared the drift of wind-sent sprayers and ultra-low volume variable sprayers using water-sensitive paper set up between fruit trees, and the experimental results showed that ultra-low volume UAVs could effectively reduce drift and improve pesticide utilization. Hunter et al. (2020) used UAV imagery for pest mapping and combined it with UAV sprayers to provide a new strategy for integrated pest management, which could improve pesticide use efficiency, reduce pesticide use, and improve the detection and control of weed escape and delay the evolution of weed resistance to herbicides. Sarri et al. (2019) collected droplets using water-sensitive paper and studied the distribution of droplet deposition and spraying efficiency in a small mountain vineyard using a spray gun, a backpack sprayer, and a UAV. The results showed that the working capacity of the UAV was twice that of the spray gun and 1.6 times that of the backpack sprayer. The coverage and deposition density of droplets were influenced by the sampling point location and the type of sprayer used. Biglia et al. (2022) investigated the effect of different UAV spraying parameters on crown spray deposition and coverage, and the experimental results showed that the flight mode had the greatest impact on spraying efficiency. Compared with the broadcast spraying mode, the strip spraying mode could increase the average crown deposition by 209% and reduce the average ground loss by 54%.

Currently, when unmanned aerial vehicles (UAVs) are used for crop spraying, the droplet size of the spray is small, which makes it susceptible to drift under the influence of environmental wind and downwash from the UAV. The larger the droplet size, the less likely it is to drift, but the deposition rate of the droplets decreases accordingly. It is often difficult to balance between a high deposition rate and low drift in UAV spraying. To address this issue, the characteristic of secondary atomization of liquid droplets after hitting a mesh can be utilized. UAVs can spray larger droplets first and then these droplets can hit the mesh and atomize into smaller droplets when approaching the target, thus achieving the advantages of low drift for larger droplets and high deposition rate for smaller droplets.

When droplets interact with a mesh, they undergo various dynamic processes such as collision, penetration, and fragmentation, which are influenced by both mesh parameters and droplet properties. Liao et al. (2022) analyzed the dynamic behavior of liquid film on stainless steel mesh surface using CFD simulations and studied the effects of different experimental conditions on the wetted area and film thickness. Ryu et al. (2017) investigated the phenomena of droplet penetration or adhesion during the process of droplet impact on mesh structures, and explored the impact factors such as droplet collision velocity, size, and mesh properties. The experimental results showed that droplets are more likely to undergo penetration and fragmentation after impact on the mesh as the droplet velocity or size increases. In addition, superhydrophobic surfaces are more likely to cause droplet penetration or rebound as a whole than ordinary surfaces. Sun et al. (2023) used high-speed cameras to study the liquid flow and rupture behavior of two liquid jets after impact on a stainless steel mesh. Kooij et al. (2019) investigated the effects of droplet properties and mesh impact velocity on the maximum spreading diameter and water droplet penetration mass after droplet impact on the mesh using experiments and simulation. Soto et al. (2018) studied the process of droplet impact on single holes and meshes and found that a conical atomization zone is formed beneath the mesh when the droplet impacts on the mesh, and the spray angle of this atomization zone increases as the velocity gradually increases. Moreover, when the velocity reaches a certain level, the spray angle tends to a fixed size that is related to the properties of the mesh, and when the velocity increases further, there is no significant change in the spray angle. Sidawi et al. (2022) investigated the spray mass fraction that penetrates through the mesh after conical spraying impacts a vertical and horizontal mesh. Moitra et al. (2021) studied the water leap phenomenon before penetration, penetration speed, and the distribution of droplets beneath the mesh after penetration by changing droplet properties (density, surface tension, and viscosity) and metal mesh properties (aperture and wire diameter).

**Figure 1:**
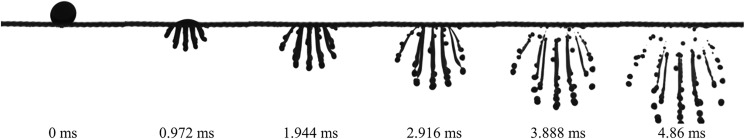
The process of liquid droplets impacting the mesh and breaking (ms= milliseconds).

Grid atomization technology uses a fine, structurally regular grid that allows droplets to pass through and break into smaller particle sizes upon impact, a characteristic that provides new and efficient system design ideas for agricultural plant protection spraying. The application of this technology can effectively improve the utilization rate of liquid solution and reduce waste. In this technique, the droplets form a jet after impacting the grid, and then the jet is broken into sub-droplets under the action of Rayleigh-Plateau instability, thus realizing the fine treatment of droplets and the optimization of spraying effect. The droplet impact grid process is shown in **Figure 1**. However, the current theoretical study mainly focuses on the case of a single droplet impacting the grid, in order to better apply the grid atomization technology in plant protection spraying, it is necessary to carry out an in-depth study on the impact of the spray composed of multiple droplets with the grid, and reasonably extend the theoretical study of a single droplet to the spray system, and further improve the theory through experimental verification.

Machine learning is a branch of artificial intelligence that can quickly discover potential patterns behind data, reduce model computation complexity, and increase model construction speed. Compared with CFD simulation, it reduces computation complexity and improves efficiency (Mosavi et al., 2018). Machine learning methods have been well applied in agriculture, biomedical and other fields (McKinney et al., 2006; Yildiz et al., 2017; Wenwen et al., 2018). Guo et al. (2020) used machine learning methods to predict the droplet size in the overlapping area of dual UAV nozzles. He et al. (2022) established quantitative models of different hollow cone nozzles’ volume median diameter (VMD) and relative span (RS) based on machine learning methods.

This study employed a combination of UAV spraying and grid atomization to examine the impact of UAV flight altitude, droplet size, and the presence of a grid on droplet deposition on target trees and downwind drift. Four machine learning methods were utilized to forecast droplet deposition and drift, resulting in the development of quantitative models. Significantly, this study represents the pioneering attempt to integrate UAVs with grid atomization to achieve a balance between high droplet deposition and minimal drift, while employing machine learning techniques for the prediction of droplet deposition and drift.

## 2 Materials and methods

### 2.1 UAV spray test method

The DJI T40 plant protection UAV used in the spray experiments features a coaxial dual-rotor design and is equipped with intelligent mapping, binocular visual perception, dual spray systems, and active phased array radar. The key parameters of the T40 are presented in **Table 1**.

**Table 1:** Parameters of DJI T40 unmanned aerial vehicle.

Key parameters of UAV	
Work box volume	40 L
Number of nozzles	2
Nozzle type	Centrifugal nozzle
Atomized particle size	50 - 300 *μ*m
Spray width	4 - 11 m
maximum flow	6 L/min * 2

Before conducting the UAV spraying experiment, a distilled water solution with a concentration of 0.5 g/L of methyl orange dye was prepared as the spray liquid. Water-sensitive paper (Chongqing LiuLiu Shanxia Plant Protection Technology Co., Ltd., with a rectangular shape of 76 mm × 26 mm), filter paper (Shanghai Peninsula Industrial Co., Ltd., with a pore size of 0.22 μm and a circular shape with a diameter of 50 mm), and nylon rope (Xiangyu Rope Net) were used as droplet collection devices. A wind speed meter (WindMaster Pro, Gill Ltd., UK) was used to monitor the environmental wind during the experiment. An oscillator and a UV spectrophotometer (UV-752, Shanghai Tianpu Analytical Instrument Co., Ltd.) were used to process the droplet collector to obtain data.

The experiment was conducted at the Citrus Research Institute in Ganzhou City, Jiangxi Province, from August 22 to 27, 2022. Prior to commencing the experiment, four air-suspended droplet samplers were designed and constructed using rigid PVC pipes with a diameter of 20 mm. These samplers were shaped as rectangular frames measuring 2 m × 1 m in height and width. To measure the drift rate of airborne droplets, samplers were positioned at distances of 3, 5, 10, and 15 m downwind from the fruit trees, starting from the UAV spraying edge. Six different heights were considered for testing, with measurements taken at varying distances from the ground. Each air-suspended droplet sampler featured a 1 m long nylon rope, with nylon ropes set at 0.3 m intervals along the sampler frame. Six nylon ropes were allocated to each sampler, resulting in a total of 24 nylon ropes used for droplet collection in a single experiment. Both ends of each nylon rope were securely fastened to the frame using 25 mm snap hooks, ensuring that the ropes remained taut and free from any bending or deformation.

**Figure 2:**
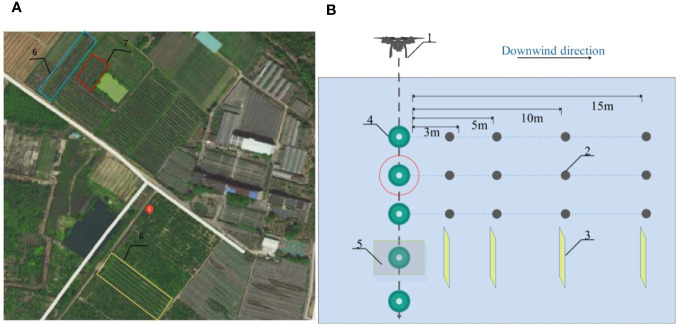
Schematic diagram of UAV test (**(A)** Three fruit tree planting areas **(B)** Experimental sampling point layout 1. Plant protection UAV; 2. Ground fog droplet collection device; 3. Aerial fog droplet sampler; 4. Citrus tree; 5.350mm aperture mesh; 6.Trellised citrus tree planting areas; 7.Dwarfed citrus tree planting areas; 8.Densely planted citrus tree planting areas).

In order to assess the dispersion pattern of drifting droplets on the ground in the downwind direction, the experimental setup followed the guidelines outlined in the ISO 22866 field testing standard for spray drift. Three Plastic Petri dishes, each with a diameter of 15 cm, were positioned at distances of 3, 5, 10, and 15 m downwind from the unmanned aerial vehicle spray swath edge. These Petri dishes were placed parallel to the flight path of the UAV. Within each dish, a water-sensitive paper and a filter paper were carefully arranged. A total of 12 Plastic Petri dishes were employed in a single testing session. The three fruit tree planting areas are shown in **Figure 2A** and the experimental layout is shown in **Figure 2B**.

For the experiment, an open flat area adjacent to a fruit tree was chosen as the designated test zone. The selected area exhibited no prominent obstacles in its immediate vicinity. To determine the drone flight route, the direction of the environmental wind was taken into consideration. The airborne droplet samplers were aligned parallel to each other, following the downwind direction and perpendicular to the wind direction. The ground drift collection device was positioned adjacent to the airborne droplet samplers, running parallel to them.

**Figure 3:**
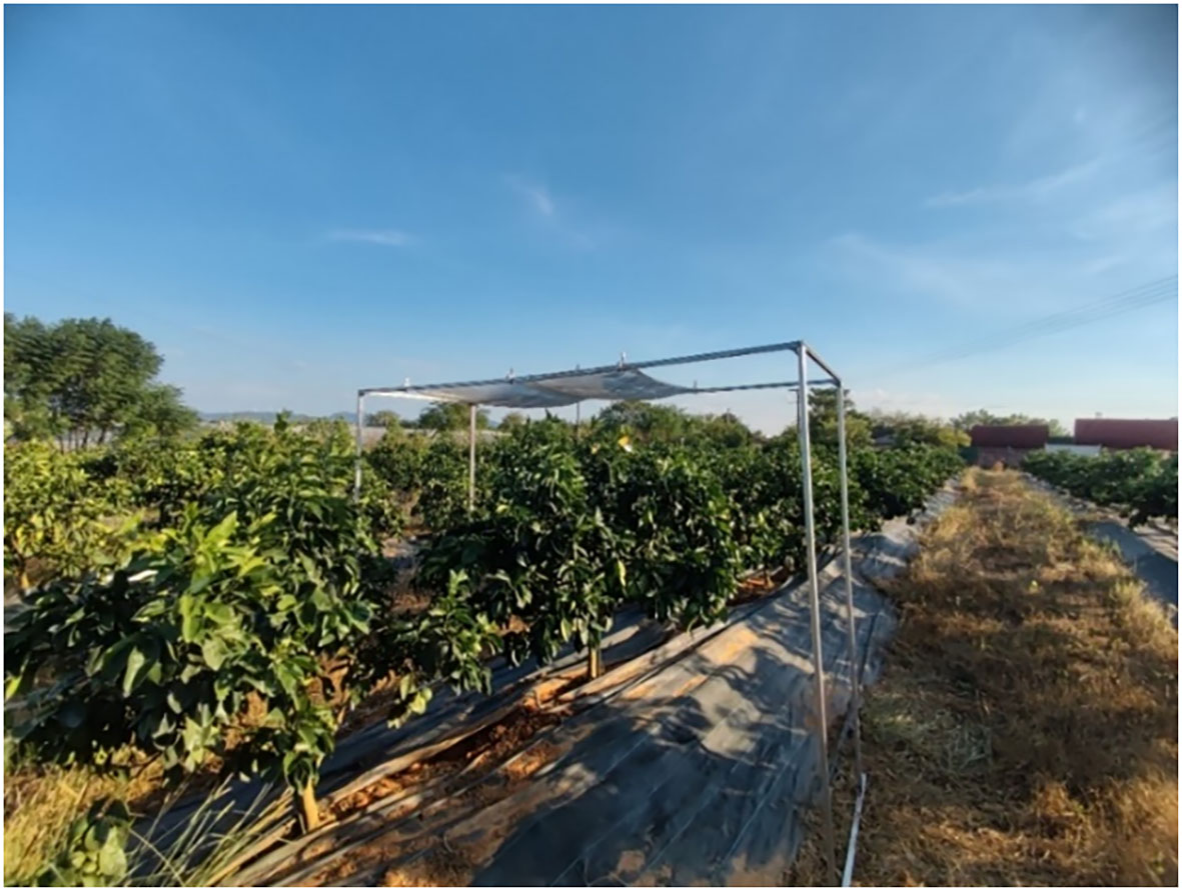
Placement of mesh.

A 3D wind speed sensor bracket was erected near the test site, ensuring it did not interfere with the spraying process. Along the UAV flight route, two trees with similar growth conditions were identified. Above one of these trees, an aluminum frame with dimensions of 2.5 m (length) × 0.7 m (width) × 2 m (height) was installed. This aluminum frame featured a mesh attached to it, characterized by an aperture size of 350 μm. Refer to **Figure 3** for a visual representation of this setup.

During the course of the experiment, two fruit trees were divided into three levels: upper, middle, and lower, with a 0.5 m gap between each level. Each level was further subdivided into three lines: front, middle, and back, with a spacing of 0.5 m. Additionally, each level was divided into left, middle, and right lines, and sampling points were established at the intersections of these lines, resulting in a total of 27 sampling points.

**Figure 4:**
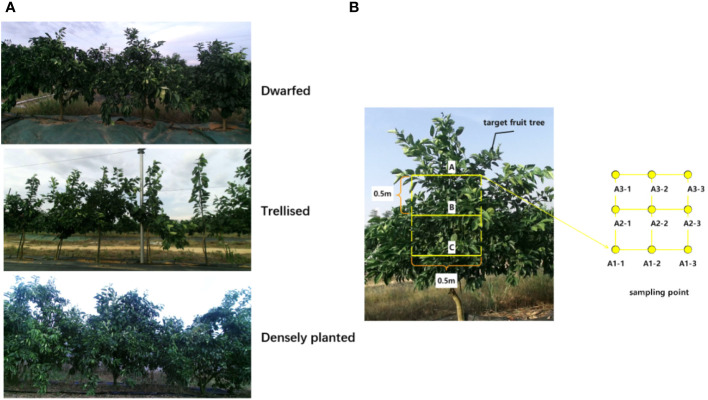
Three forms of trees and target fruit tree sampling site layout (**(A)** Three tree-shaped fruit trees **(B)** Sampling point arrangement of target fruit trees).

The three levels were labeled as A, B, and C, with A1, A2, and A3 representing the front, middle, and back positions of level A, respectively. The left, middle, and right points were denoted as A1-1, A1-2, A1-3, A2-1, A2-2, A2-3, A3-1, A3-2, and A3-3. At each sampling point, two water-sensitive papers and two filter papers were fixed using paperclips on the front and back sides, without overlapping. The morphology of the three fruit trees is shown in **Figure 4A**, and the water-sensitive paper arrangement of the target fruit trees is shown in **Figure 4B**.

In accordance with the ISO 22866 standard, the acceptable wind speed for lateral environmental wind drift tests ranged from 1.0 to 5.0 m/s (at a height of 2 m), with a permissible wind direction angle deviation of 90° ± 30° from the flight route. The flow rate of the plant protection UAV was calibrated, and real-time monitoring of wind speed and direction was carried out. Once the wind speed and direction met the standard requirements and remained stable for one minute, a methylene orange solution was introduced into the tank.

The experimental trees comprised three types of citrus trees, namely dwarf, hedge-style, and dense planting. The flight path of the UAV was pre-determined, with the UAV flying vertically over the trees at a height of 2, 3, or 4 m. The UAV maintained a speed of 2 m/s while spraying pesticide at a rate of 50 L/hm2. The nozzle sprayed droplets with sizes of very coarse, medium, and very fine, according to the instructions in the plant protection UAV manual, the three particle sizes are 140μm, 100μm and 60μm respectively. The UAV followed the designated tree route, conducting spraying during the flight. After completing one spraying operation, the UAV avoided the sampling point area to prevent interference with the data. It then returned to the takeoff point.

Once the water-sensitive papers, filter papers, and nylon ropes at the sampling points were completely dry, they were collected using forceps and immediately placed in self-sealing plastic bags sized 22 cm × 15 cm to avoid cross-contamination between samples. Each experimental condition was repeated three times, and the final data was averaged. After concluding the full day of experiments, all samples (including water-sensitive papers, filter papers, and nylon ropes) were transported to a cool, dark storage location for further analysis and uniform processing.

### 2.2 Machine learning methods

Prior to training the quantitative model, the dataset in this study was divided into training and prediction sets at a ratio of 3:1, ensuring the model’s robustness. To explore the meaningful relationship between the independent and dependent variables, a one-way ANOVA analysis was conducted on the data using SPSS software.

The study employed four primary machine learning algorithms, which are as follows:

(1) Multi-dimensional non-linear regression analysis was conducted using the REGRESS function in MATLAB software, which employs orthogonal least squares method and has been widely used in biomedical and financial fields (Arnisigo et al., 2008; Steed et al., 2009; Lü et al., 2014). The REGRESS function calculates the estimated ratio of the observation value residuals to their standard deviation using orthogonal least squares method. The resulting value is t-distributed with a certain degree of freedom, and the function returns the offset of the t-distribution confidence interval with the residuals as the center (Hoaglin and Kempthorne, 1986). The significance of the model was evaluated using the F statistic, with a significance level of 0.05 in this study, and the confidence interval for the estimated values was set at 95%.
(2) The Back Propagation Neural Network (BPNN) belongs to the multi-layer feedback network category. It uses the Back Propagation algorithm for training, which does not require an explicit functional relationship between input and output vectors before training. The algorithm uses gradient descent to iteratively adjust the biases and weights of each layer in the network to minimize the error between predicted and expected outputs. During the Back Propagation process, the network updates the weight values of each neuron to adjust the parameters of the neural network, improving its predictive ability. When the error reaches its minimum value, the calculated output of the input value is closest to the expected output, which is used as the predicted value.
(3) Extreme Learning Machine (ELM) is a type of feedforward neural network that does not require gradient-based backpropagation to adjust weights. Instead, ELM sets the weight values using the Moore-Penrose generalized inverse matrix and has only one hidden layer, resulting in extremely fast computation speeds (Huang et al., 2006).
(4) The Radial Basis Function Neural Network (RBFNN) is a feedforward neural network with a 3-layer structure, consisting of an input layer, a hidden layer with radial basis functions (RBF) as activation functions, and an output layer. This machine learning method is widely used for classification and regression analysis due to its fast training speed and strong generalization ability (Schalkoff, 1997).

### 2.3 Performance evaluation

#### 2.3.1 Droplet deposition rate/drift rate analysis method

The water-sensitive papers from the samples were scanned using a scanner at a grayscale resolution of 600 dpi. The obtained images were subsequently processed using ImageJ software to determine the density and coverage of the spray deposition. As for the filter paper samples, they were placed in plastic self-sealing bags along with 50 mL of distilled water. These bags were subjected to oscillation at a frequency of 200 r/min for a duration of 30 minutes to extract the chemicals. The resulting eluates were then analyzed using a UV-Vis spectrophotometer to measure the absorbance specifically at a wavelength of 465 nm. Based on these measurements, the deposition and drift amounts were calculated.

To measure the deposition and drift of droplets on filter paper and nylon rope, a UV-752 UV/visible spectrophotometer (Shanghai Tiantu Analytical Instrument Co., Ltd.) was used to calibrate the concentration-absorbance relationship. A linear regression equation was derived through linear fitting, correlating the methyl orange concentration (a) in mg/L with the absorbance value (b) of the test solution. For the filter paper, 10 mL of distilled water was added to the self-sealing bag containing the sample. Similarly, for the nylon rope, 50 mL of distilled water was added to its respective self-sealing bag. These bags were then oscillated on an oscillator at a frequency of 200 r/min for a duration of 30 minutes. Subsequently, 3 mL of eluent was extracted using a pipette, and its absorbance value was measured at a detection wavelength of 465 nm using the UV-752 UV/visible spectrophotometer. The methyl orange concentration was determined by utilizing the previously established regression curve. Finally, the deposition rate of droplets was calculated based on **Formulas 1**, 2.


(1) $$\beta =\frac{Ce_{1}\times V}{Ce_{2}\times S}$$

(2) $$\beta_{dep\%}=\frac{\beta }{\beta_{v}}*100\%$$

Where *β* is the deposition of droplets per unit area(*μ*L/cm^2^); *Ce*_1_ is the concentration of methyl orange in the elution solution(mg/L); *V* is the volume of elution solution added(*μ*L); *Ce*_2_ is the concentration of methyl orange in the spray solution(mg/L); *S* is the area of the droplet collector(cm^2^); *β_dep%_* is the deposition/drift rate(%); *β*_v_ is the application rate(L/m^2^).

#### 2.3.2 Analysis method of droplet ground drift rate

In this study, the Average Average Drift Rate (*AADR*) is used to indicate the extent of droplet drift. *AADR* represents the average of all data means at each downwind distance during each spray operation. The calculation is shown in **Formulas 3**:


(3) $$AADR=\frac{\sum_{\text{i}=1}^{\text{n}}{\overset{¯}{\text{β}}}_{\text{dep}\%\text{i}}}{\text{n}}$$

Where ${\bar{\beta }}_{dep\%i}$ is the mean drift rate of the i-th group at downwind distance; *n* represents the number of sampling groups at different downwind distances.

According to ISO 22866 standard, the percentage of cumulative drift of droplets $\beta_{total\%}$ from the edge of the spray plume to a downwind distance *x*, relative to the total drift, is defined as the cumulative drift ratio of droplets $\beta_{cum\%}$ . The downwind distance at which $\beta_{cum\%}$ reaches 90% is defined as the 90% cumulative drift distance $x_{90\%}$ . The calculation method is shown in Formulas 4, 5:


(4) $$\beta_{cum\%}=\frac{\int_{1}^{\text{x}}{\text{β}}_{\text{dep}\%}\left(\text{x}\right)\text{dx}}{{\text{β}}_{\text{total}\%}}\times 100\%$$

(5) $$\beta_{total\%}=\int_{1}^{x_{m}}\beta_{dep\%}\left(x\right)dx$$

Where $\beta_{dep\%}\left(x\right)$ represents the drift rate at a downwind distance of *x*(%); *x_m_* represents the distance from the edge of the spray plume to the farthest ground-level droplet collector(m).

#### 2.3.3 Analysis method of droplet drift rate in air

*h_r_* represents the relative feature height, indicating the relative position of the center of droplet drift distribution on the droplet collection framework. A higher relative feature height indicates a greater extent of airborne droplet drift at the current downwind distance. The calculation method for the relative feature height is shown in Formula 6:


(6) $$h_{r}=\frac{s\cdot \sum_{i=1}^{{\text{n}}_{\text{p}}}{\text{β}}_{\text{dep}\%\text{i}}{\text{h}}_{\text{i}}}{{\text{h}}_{\text{max}}}$$

Where *s* represents the distance between each nylon rope in the airborne droplet sampler (0.3 m); *n_p_* represents the number of nylon ropes on each droplet collection device ( $n_{p}=10$ ); $\beta_{dep\%i}$ represents the drift rate of droplets on the i-th nylon rope; *h_i_* represents the height of the i-th nylon rope in meters; *h_max_* represents the height of the highest nylon rope ( $h_{\max}=1.8m$ ).

#### 2.3.4 Quantitative model analysis method

This study evaluates the performance of various machine learning quantitative models using the Coefficient of Determination (R^2^) and Root Mean Squared Error (RMSE). R^2^, also known as the multiple correlation coefficient, is defined as the ratio of variances in the regression model. This definition makes it a measure of the success rate of predicting the dependent variable from the independent variables (Nagelkerke, 1991). R_t_^2^ and R_p_^2^ are the determination coefficients for the training set and the prediction set, respectively, indicating the accuracy of the predictive model. RMSE is used to measure the error of the model, including the Root Mean Squared Error of the training set (RMSET) and the Root Mean Squared Error of the prediction set (RMSEP). A smaller RMSE indicates better performance and higher accuracy of the model. R2 and RMSE can be calculated using Formulas 7, 8.


(7) $$R^{2}={\left(\frac{\sum_{\text{i}=1}^{\text{N}}\left({\hat{\text{y}}}_{\text{i}}-\overset{¯}{\hat{\text{y}}}\right)\left({\text{y}}_{\text{i}}-\overset{¯}{\text{y}}\right)}{\sqrt{\sum_{\text{i}=1}^{\text{N}}{\left({\hat{\text{y}}}_{\text{i}}-\overset{¯}{\hat{\text{y}}}\right)}^{2}{\left({\text{y}}_{\text{i}}-\overset{¯}{\text{y}}\right)}^{2}}}\right)}^{2}$$

(8) $$RMSE=\sqrt{\frac{\sum_{i=1}^{N}{\left(y_{i}-{\hat{y}}_{i}\right)}^{2}}{N-1}}$$

where $y_{i}$ and ${\hat{y}}_{i}$ are the measured value and the predicted value of the i-th sample, $\bar{y}$ and $\bar{\hat{y}}$ are the average values of measured and predicted values, respectively; N is the number of samples.

## 3 Results and discussion

### 3.1 Droplet coverage and deposition density

**Figure 5:**
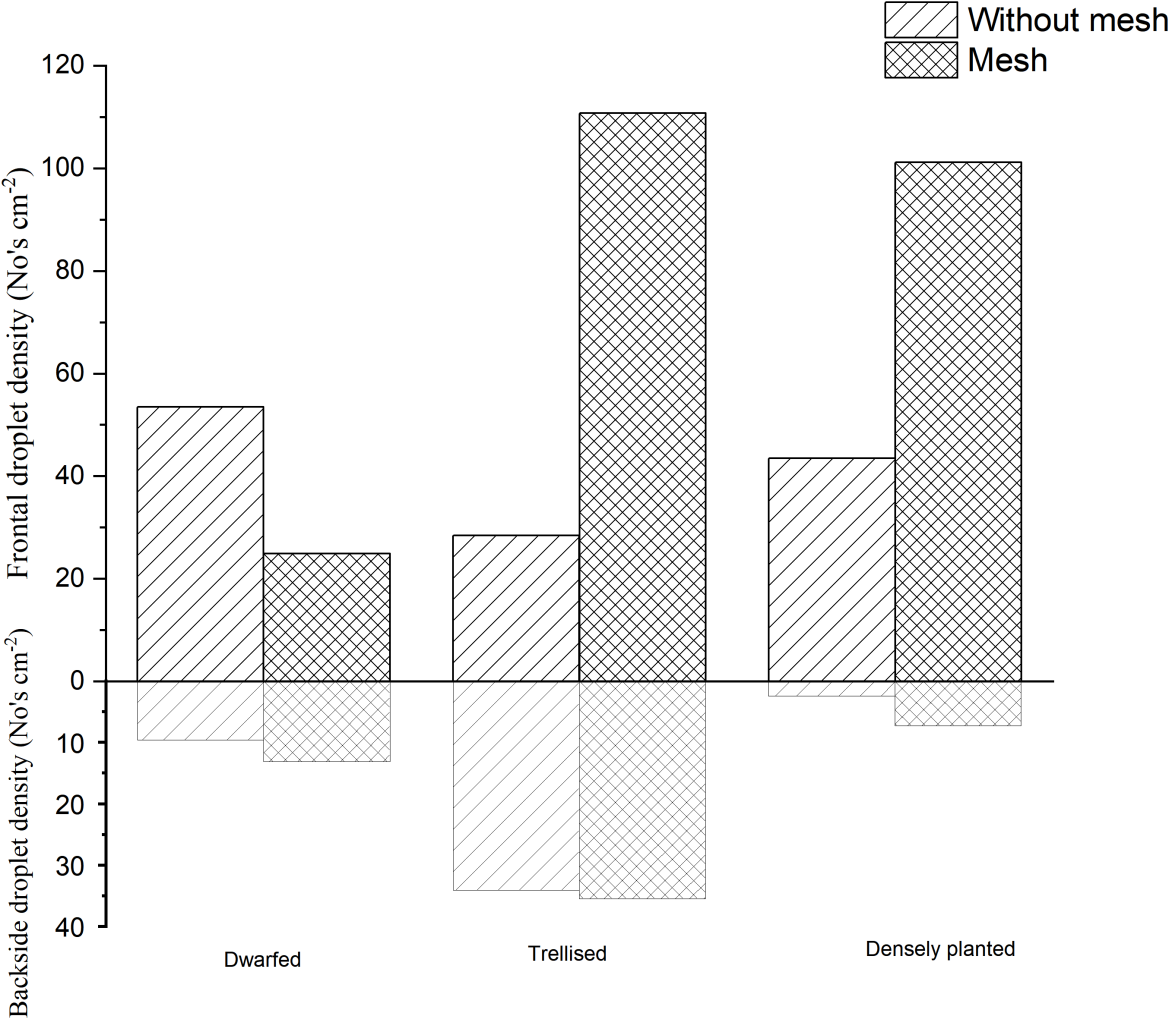
Coverage of droplets in different tree forms.

**Figure 6:**
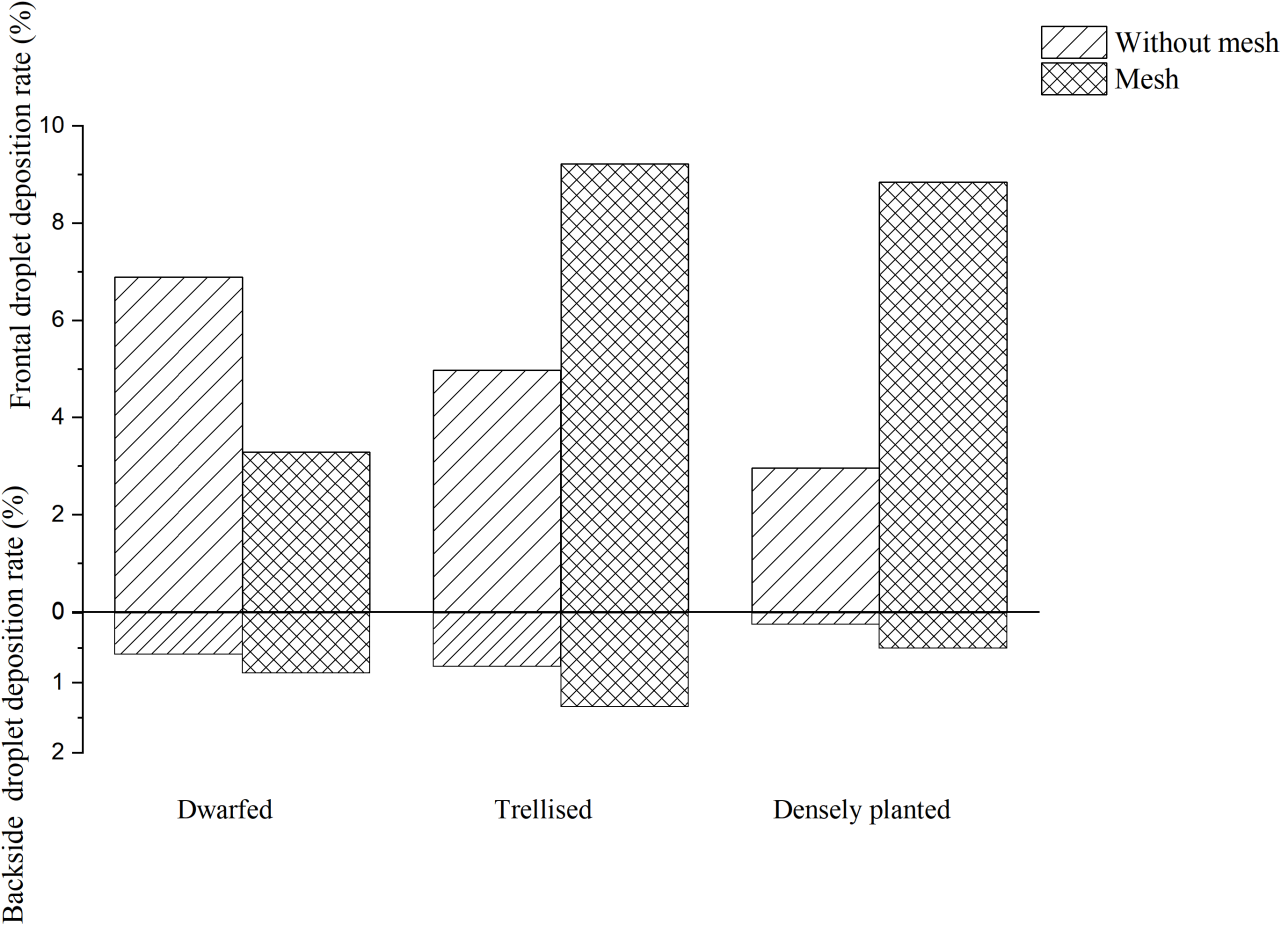
Droplet density of droplets with different tree forms.

The images of water-sensitive paper were processed using ImageJ software to quantify the coverage and deposition density of droplets at each sampling point. Subsequently, the average droplet coverage and deposition density on the front and back surfaces of leaves from various tree forms, with and without a grid, were calculated. The results are presented in **Figures 5**, **6**.

The analysis of **Figure 5** reveals that, in the absence of grid placement, the average droplet coverage on the front side of the fruit trees across the three tree forms ranged from 2.96% to 6.89%, while on the reverse side it varied between 0.16% and 0.77%. With grid placement, the average droplet coverage on the front side of the fruit trees of the three tree forms ranged between 3.29% and 9.22%, and between 0.5% and 1.34% on the reverse side. The introduction of the grid led to an improvement in droplet coverage on the reverse side for all three tree forms, resulting in an increase of 0.27% for dwarfed citrus trees, 0.57% for trellised citrus trees, and 0.34% for densely planted types. Conversely, the grid placement resulted in a decrease in droplet coverage by 2.5% on the front side of dwarfed citrus trees, while increasing droplet coverage by 4.24% on trellised citrus trees, and exhibiting the most significant increase of 5.98% on the densely planted citrus trees.

The analysis of **Figure 6** reveals that, in the absence of the grid, the average droplet deposition density on the front side of the three forms of tree ranged from 28.45 drops per square centimeter to 53.45 drops per square centimeter, while on the reverse side it varied between 2.46 drops per square centimeter and 34.14 drops per square centimeter. With the grid placement, the average droplet deposition density on the front side of the three tree forms ranged between 24.9 drops per square centimeter and 110.71 drops per square centimeter, and between 7.29 drops per square centimeter and 35.48 drops per square centimeter on the reverse side. The introduction of the grid led to an improvement in the mean droplet deposition density on the reverse side of the three tree forms, although the improvement was not statistically significant. However, the addition of grids had a significant impact on the frontal droplet deposition density for trellised and the densely planted citrus trees. The frontal droplet deposition density increased by 82.26 drops per square centimeter (289%) for the densely planted citrus trees and by 57.62 drops per square centimeter (132%) for the densely planted citrus trees. In contrast, the mean frontal droplet deposition density of dwarfed citrus trees decreased by 28.55 drops per square centimeter due to the addition of the grid.

Increasing the grid has shown clear improvements in droplet deposition density and coverage on the front side of trellised and the densely planted citrus trees. However, for dwarfed citrus trees, the addition of the grid resulted in a decrease in droplet deposition density and coverage on the front side of the tree. This can be attributed to the fixed height of the grid, which causes dwarfed citrus trees to be farther away from the grid compared to the other two types of trees. The droplets produced by the grid undergo a second atomization, resulting in smaller particle sizes. These smaller droplets are more susceptible to drift during their movement towards the dwarfed citrus trees. As a result, the droplets may not reach the intended target as effectively, leading to a decrease in droplet deposition density and coverage on the front side of the dwarfed citrus trees.

### 3.2 Droplet deposition rate

**Figure 7:**
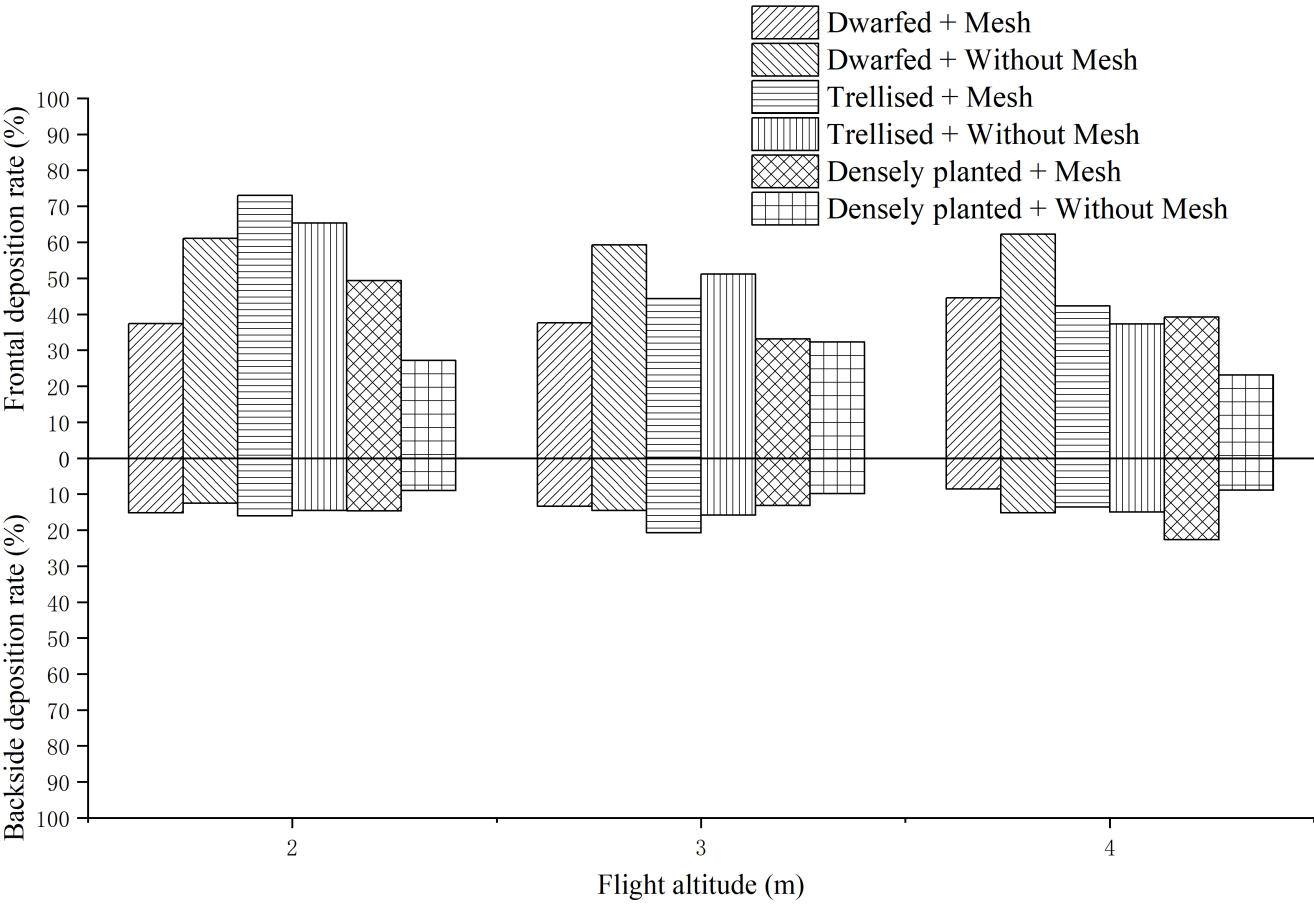
Droplet deposition rate at different flight altitudes.

The deposition rates of droplets on the adaxial and abaxial surfaces of three citrus tree leaf forms at different flight heights were determined by measuring filter papers, as shown in **Figure 7**.

Based on the observations from **Figure 7**, it can be noted that the deposition rates of droplets on the upper (adaxial) surface of the leaves range from 23% to 73%, while the deposition rates on the lower (abaxial) surface range from 8.57% to 22.61%. As the flight height of the UAV increases, the deposition rate on the adaxial surface gradually decreases. The maximum deposition rate of 52.21% is observed at a flight height of 2 m, which is higher by 9.26 and 10.76 percentage points compared to the rates at 3 m (42.95%) and 4 m (41.45%), respectively. On the other hand, the flight height has a lesser impact on the deposition rate on the abaxial surface, as the rates remain relatively consistent across the three flight heights.

Among the three forms of citrus trees, the densely planted trees exhibit the lowest deposition rate on the adaxial surface, with an average of 34.04%. In contrast, the average deposition rates on the adaxial surface of dwarfed and trellised citrus trees exceed 50%, measuring at 50.34% and 52.22%, respectively. This indicates that the deposition rates on dwarfed and trellised citrus trees are higher compared to traditionally densely planted citrus trees. This can be attributed to the smaller canopy size of dwarfed and trellised citrus trees, which allows droplets to penetrate the canopy and deposit on the lower parts.

The average deposition rates of droplets with and without the placement of grids on citrus trees are 44.51% and 46.55%, respectively. This suggests that the placement of grids has minimal effect on the deposition rate of droplets on citrus trees, resulting in only a 2.04 percentage point decrease on average.

**Figure 8:**
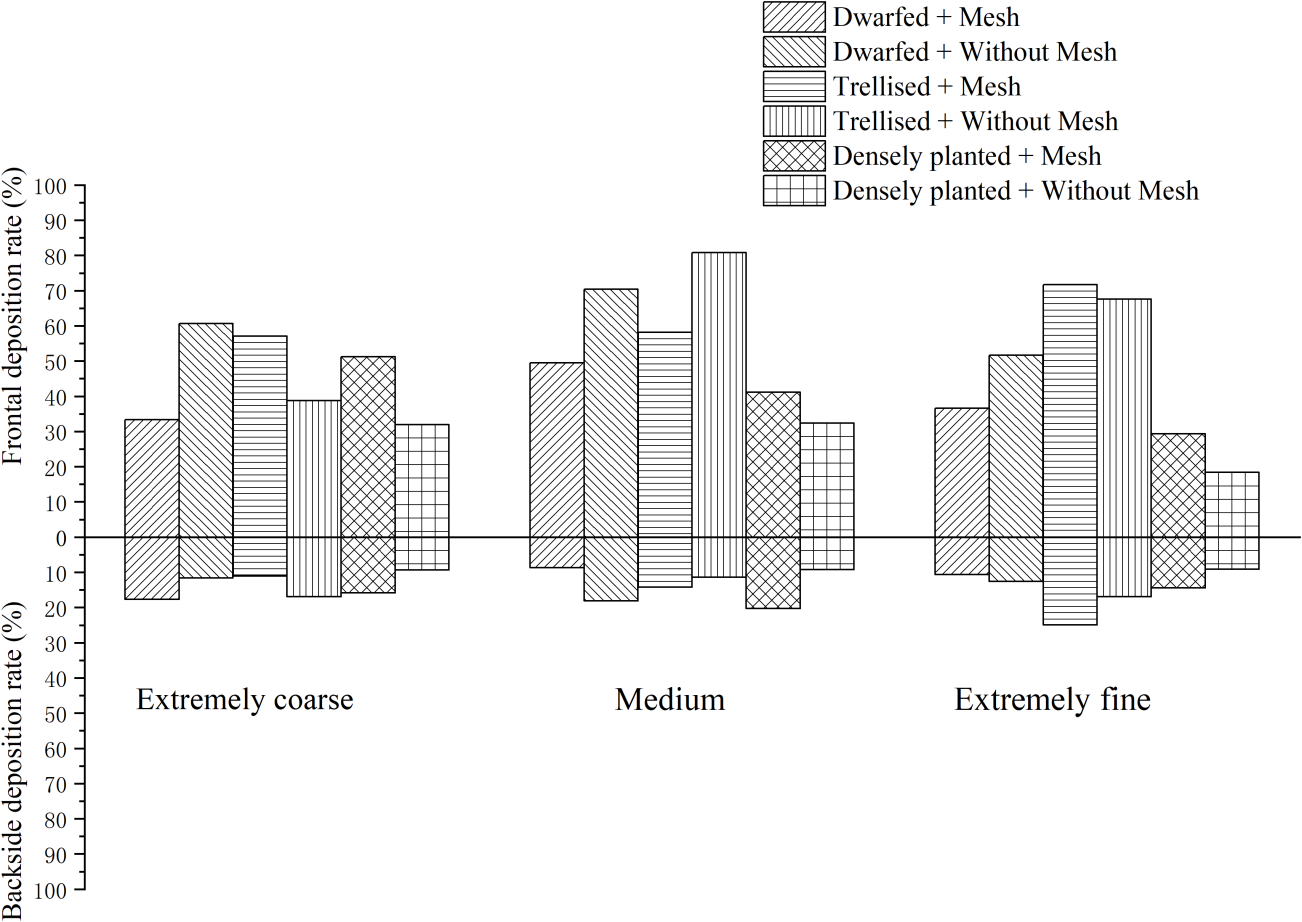
Droplet deposition rate under different droplet sizes.

The deposition rates of droplets on the adaxial and abaxial surfaces of three citrus tree leaf forms at different droplet sizes, as measured using filter papers on citrus trees, are shown in **Figure 8**.

Based on the observations from **Figure 8**, it can be noted that among the three different sizes of droplets, the deposition rate on the upper (adaxial) surface is highest for medium-sized droplets, averaging at 55.39%. The deposition rates for very coarse and very fine droplets are comparatively lower, measuring at 45.48% and 45.86%, respectively. This discrepancy in deposition rates can be attributed to certain factors.

Very coarse droplets have a tendency to rebound from the leaf surface, making it challenging for them to adhere effectively. As a result, their deposition rates are lower compared to medium-sized droplets. On the other hand, very fine droplets are more susceptible to environmental winds and the downwash airflow generated by the UAV. These factors contribute to the drift of the fine droplets, reducing their ability to deposit on the leaf surface and resulting in lower deposition rates.

**Figure 9:**
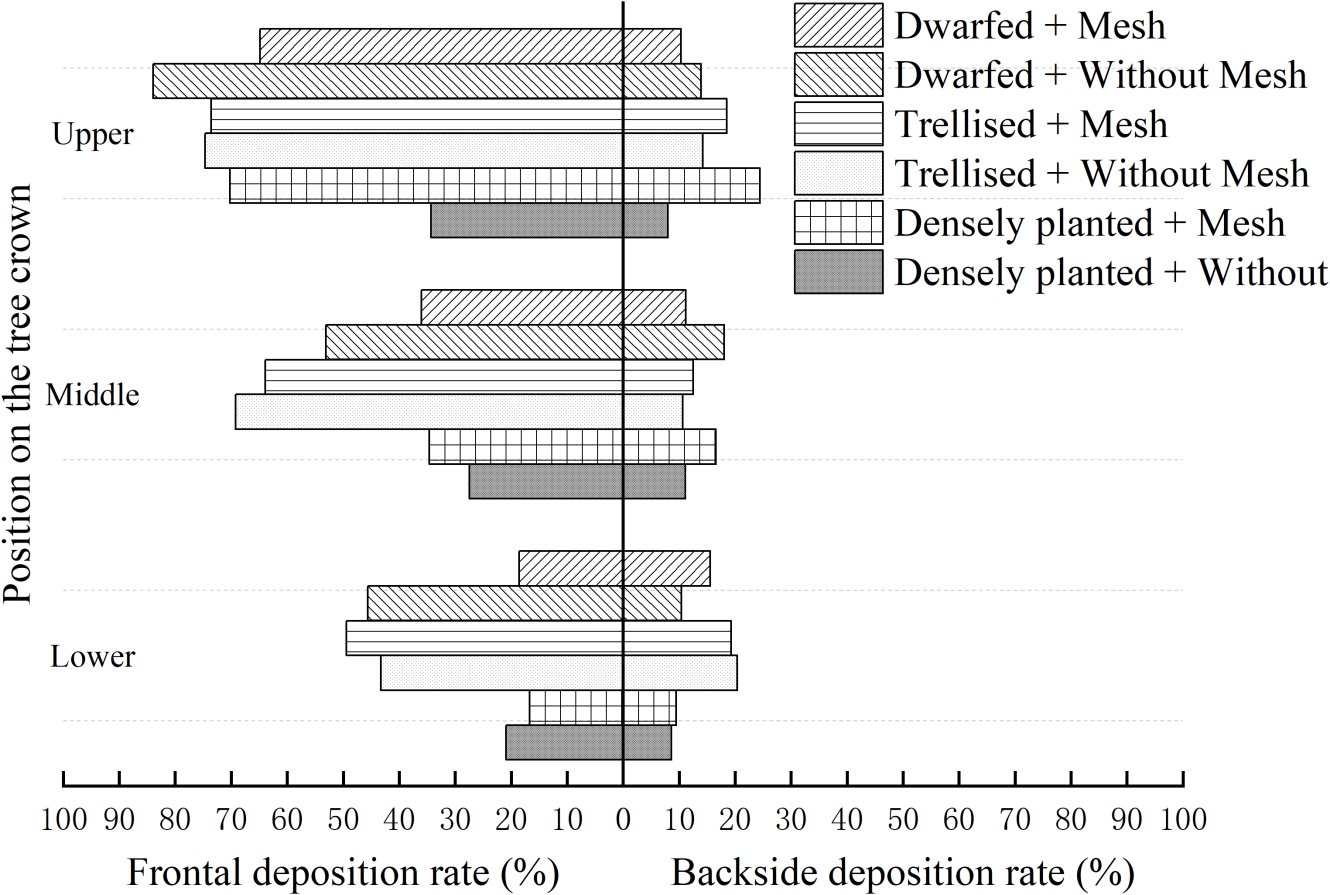
Droplet deposition rate in upper, middle and lower layers of tree canopy.

The deposition rates of droplets on the adaxial and abaxial surfaces of the upper, middle, and lower layers of leaves in three citrus tree forms, as measured using filter papers on citrus trees, are shown in **Figure 9**.

Based on the observations from **Figure 9**, it can be noted that the deposition rates of droplets on the upper (adaxial), middle, and lower layers of the citrus tree canopy range from 16.68% to 83.89%. On the lower (abaxial) surface, the deposition rates range from 8.60% to 24.42%.

The deposition rate on the adaxial surface gradually decreases as we move from the upper layer to the lower layer of the canopy. In the upper layer, the average deposition rate is 66.91%, which is higher by 19.52 and 34.48 percentage points compared to the rates in the middle layer (47.39%) and lower layer (32.43%), respectively. However, the deposition rates on the abaxial surface show little variation across the different layers of the canopy.

Among the layers of the canopy, the disparity in deposition rates between the upper (adaxial) and lower (abaxial) surfaces is most pronounced in the upper layer. The deposition rate on the adaxial surface is 4.5 times higher than that on the abaxial surface in the upper layer, while in the middle and lower layers, this ratio decreases to 3.5 and 2.3 times higher, respectively. As the height decreases, the difference in deposition rates between the adaxial and abaxial surfaces also decreases.

In the presence of grids, the deposition rate on the adaxial surface is 3.1 times higher than that on the abaxial surface, whereas in their absence, this ratio increases to 3.9 times higher. This indicates that the placement of grids on citrus trees can enhance the deposition of droplets on the abaxial surface, consequently increasing the overall deposition rate. The grids facilitate this improvement by causing secondary atomization of droplets upon impact. This process leads to reduced movement velocity and droplet size, thereby enhancing the adherence of droplets to the abaxial surface of the leaves.

### 3.3 Ground droplet drift

**Figure 10:**
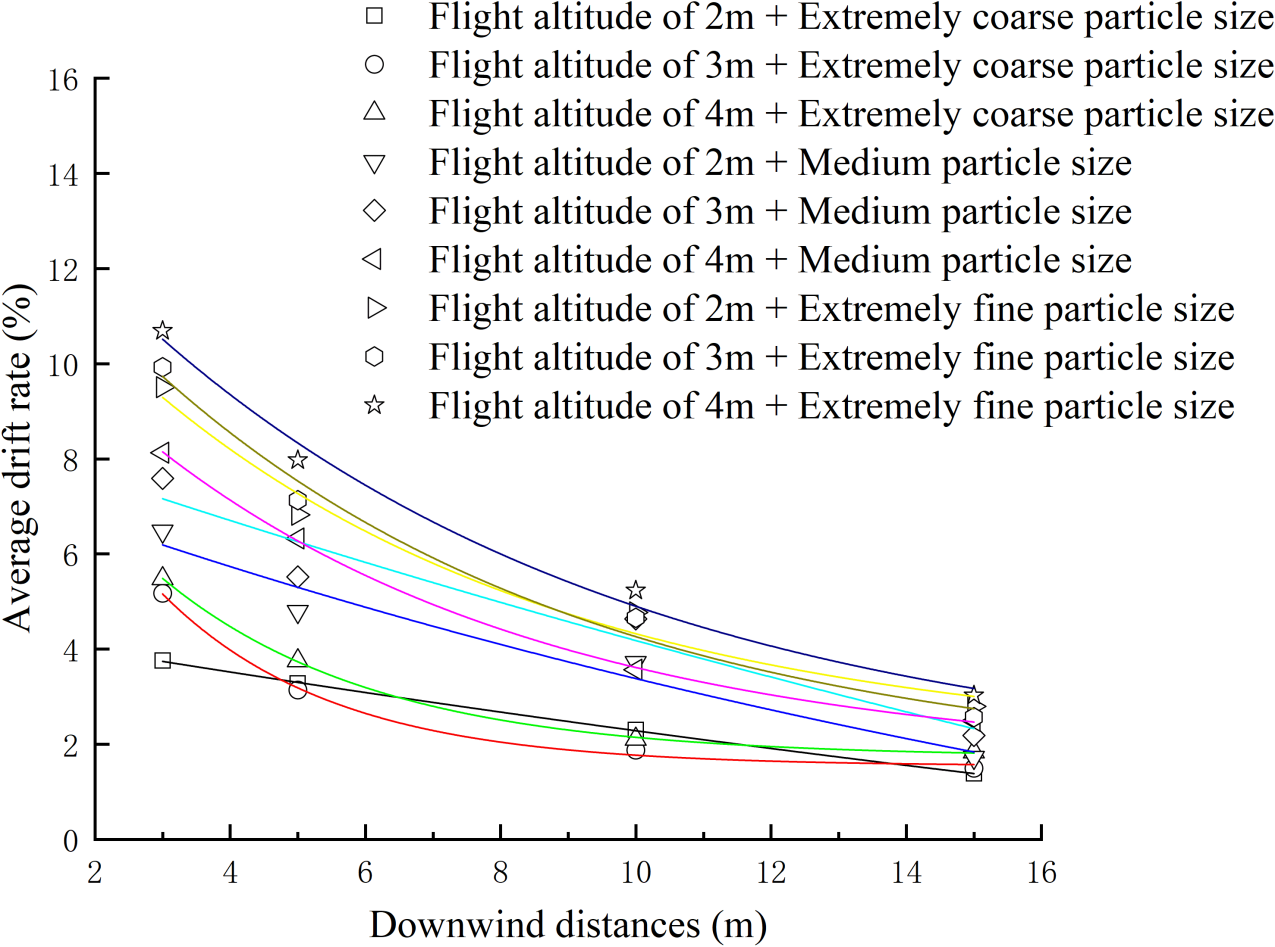
Variation of average drift rate of ground sampling points with downwind distance and its exponential function regression curve under various test conditions.

The non-linear regression analysis of the mean drift rate was conducted using Origin 2018 software, and the fitted curve was plotted as shown in **Figure 10**. The analysis aimed to examine the effects of changes in downwind distance, different flight heights, and droplet sizes on the mean drift rate.

Based on the observations from **Figure 10**, it can be noted that the drift rate of sprayed droplets from the UAV gradually diminishes as it extends to a distance of 15 m. However, under certain conditions, the drift rate of droplets at the 15 m mark can still exceed 3%. This indicates that the actual drift distance of droplets at this point is greater than 15 m. The most rapid decrease in drift rate occurs within the range of 3 to 5 m. The drift rate of UAV spraying exhibits an exponential relationship with the downwind distance, with a decrease in drift rate as the downwind distance increases.

**Figure 11:**
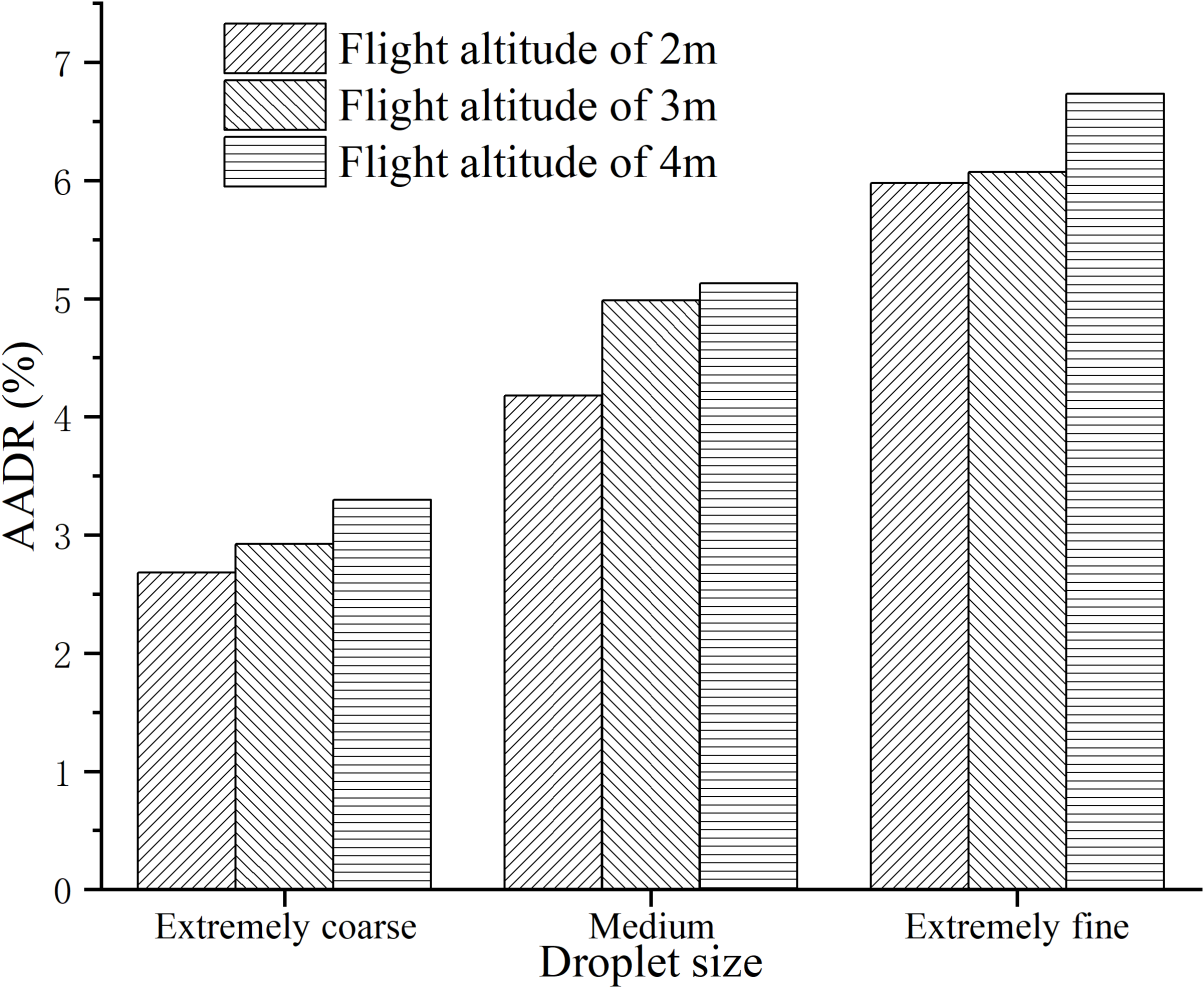
*AADR* of ground droplets under different operating parameters.

**Figure 11** displays the Average Absolute Drift Rate (*AADR*) of droplets under different operational parameters of the plant protection UAV.

Based on the observations from **Figure 11**, it is evident that both droplet size and UAV flight height have a significant impact on the Average Absolute Drift Rate (*AADR*) of droplets. The *AADR* decreases as the droplet size increases and increases with higher UAV flight heights. The lowest *AADR*, at 2.68%, is observed with a UAV flight height of 2 meters and the droplet size classified as “coarse.” In contrast, the highest *AADR* is 6.73%, representing an increase of 4.05 percentage points.

**Figure 12:**
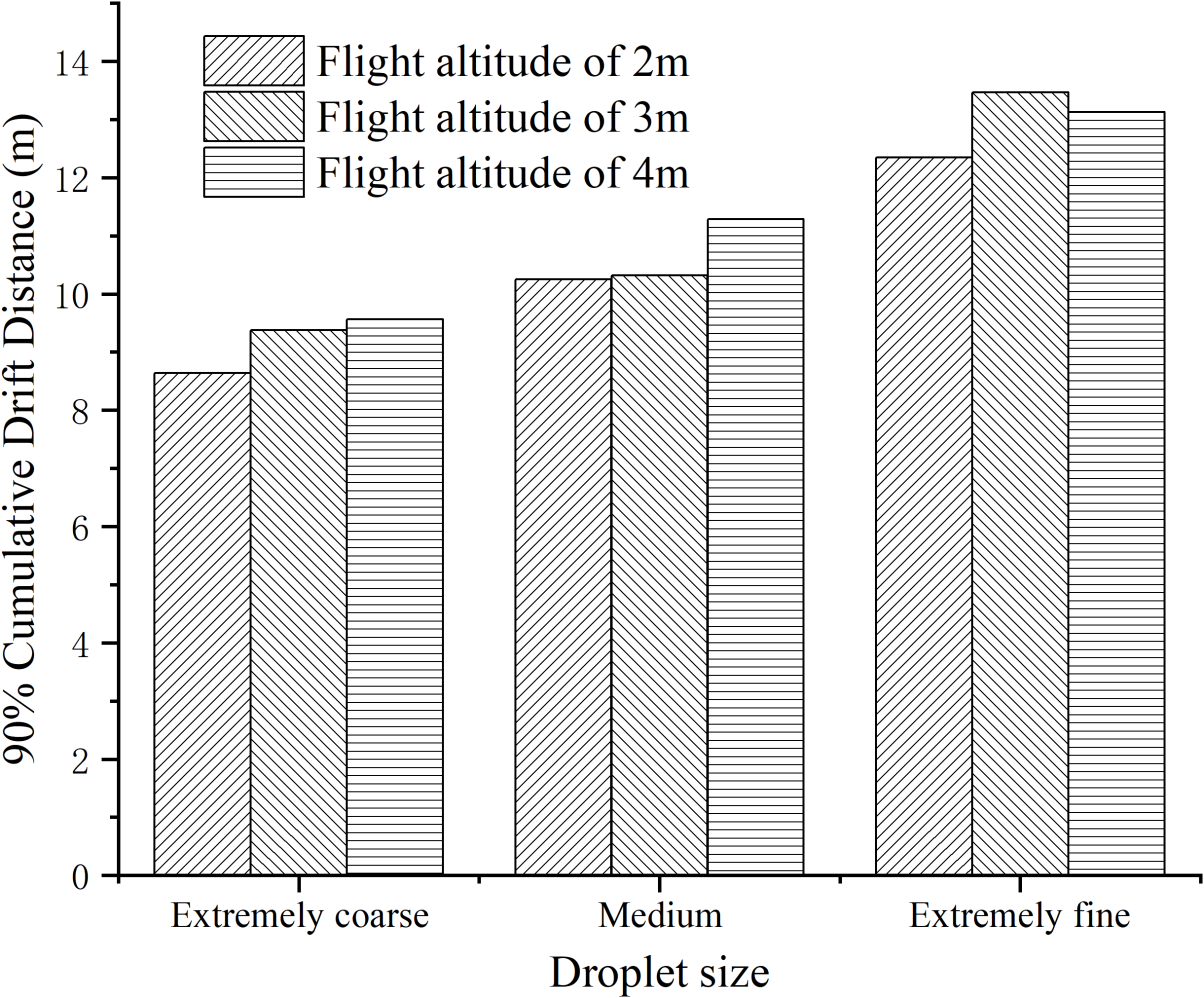
90% cumulative drift distance of ground droplets under different operating parameters.

**Figure 12** displays the 90% cumulative drift distance of droplets under different operational parameters of the plant protection UAV.

Based on the observations from **Figure 12**, it can be noted that the 90% Cumulative Drift Distance (CDD) ranges from 8.6 to 13.5 meters. The 90% CDD increases as the droplet size decreases, indicating that larger droplets result in a decrease in the 90% CDD by 2 to 5 meters. In contrast, the UAV flight height has minimal influence on the 90% CDD. For coarse or medium droplet sizes, the 90% CDD increases with increasing flight height, reaching its maximum at 4 meters. However, for extremely fine droplets, the 90% CDD is highest at a flight height of 3 meters. When the droplet size is kept constant, the difference in the 90% CDD is within 1.2 meters.

### 3.4 Droplet drift in the air

**Figure 13:**
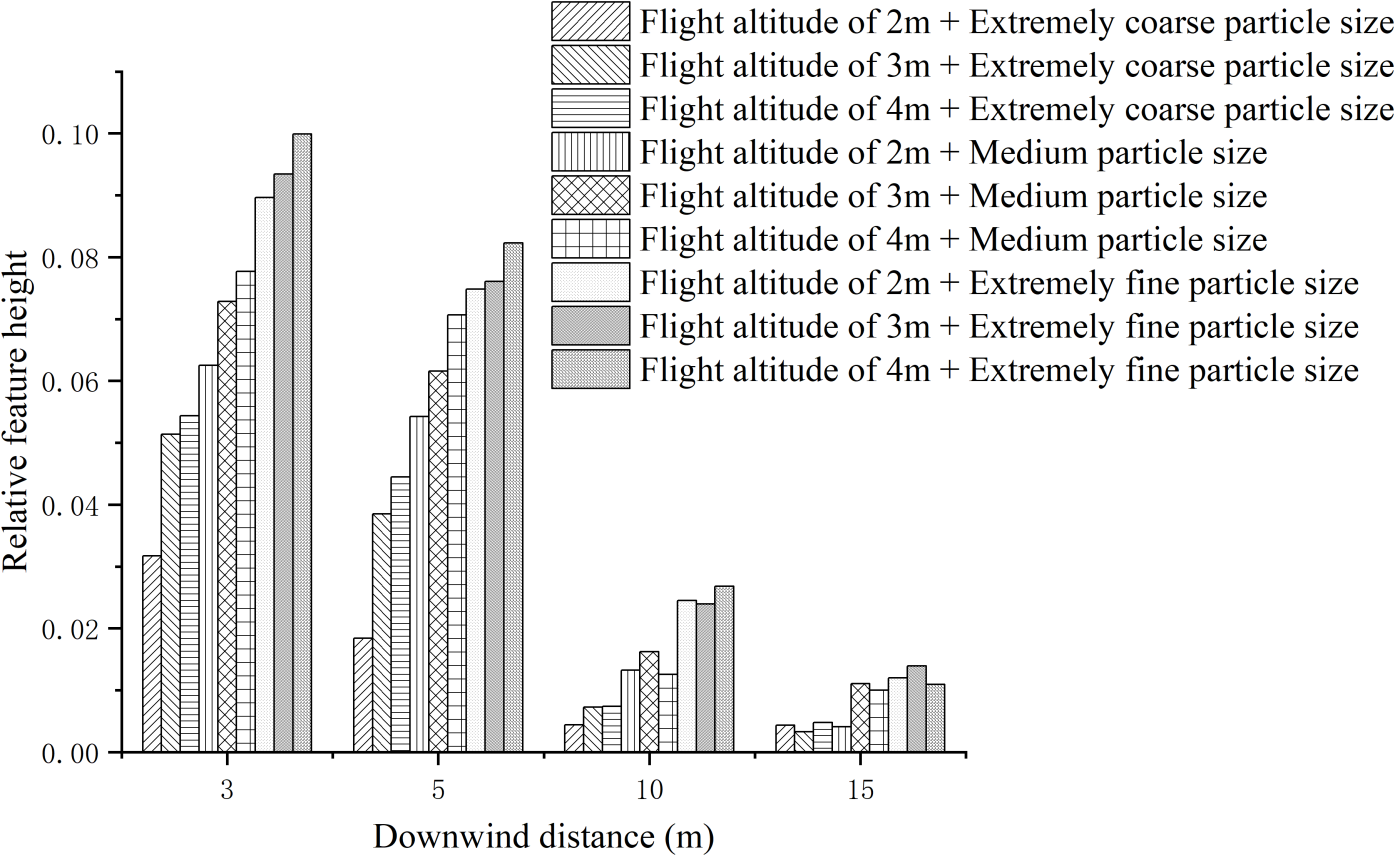
Relative characteristic height of droplets at different downwind distances and different test parameters.

The results obtained from the calculation of relative feature heights based on the vertical drift rates of droplets at different downwind distances are presented in **Figure 13**. It can be observed that the relative feature height decreases as the downwind distance increases. At downwind distances of 3m and 5m, the relative feature height increases with higher flight heights and smaller droplet sizes, indicating an increase in droplet drift. This observation aligns with the analysis of ground-level droplet drift.

At downwind distances of 10m and 15m, the relative feature height still increases with decreasing droplet size, while the flight height has minimal influence on the relative feature height at this point.

### 3.5 Prediction of droplet deposition rate

In the experiment conducted on the DJI T40 plant protection UAV, significance analysis was performed to examine the influence of flight altitude, droplet size, and sampling height on the droplet deposition rate. The results of the analysis are presented in **Table 2**.

**Table 2:** Droplet deposition rates at different flight altitudes, droplet sizes, sampling points.

		Deposition rate
Flight altitude/m	2	0.373 ± 0.175^a^
	3	0.376 ± 0.304^a^
	4	0.445 ± 0.357^a^
Droplet size/μm	60	0.367 ± 0.173^a^
	100	0.495 ± 0.379^a^
	140	0.333 ± 0.253^a^
Sampling point height/m	1	0.186 ± 0.124^a^
	1.5	0.360 ± 0.215^a^
	2	0.648 ± 0.263^b^

The deposition rate data in the table is the mean ± standard deviation, dimensionless; The same small letters indicate that there is no significant difference in droplet deposition rate under different droplet sizes, and the significance level setting p=0.05.

Comparing different flight altitudes on the droplet deposition rate, three flight altitudes were selected for analysis. According to **Table 2**, there is no significant relationship observed between flight altitude and the droplet deposition rate.

Analyzing the impact of droplet size on the droplet deposition rate, three different droplet sizes were analyzed. The results in **Table 2** indicate that there is no significant relationship between droplet size and the droplet deposition rate.

When examining the effects of different sampling heights on the droplet deposition rate, three specific sampling heights were chosen. Based on the results presented in **Table 2**, a significant relationship is observed between the droplet deposition rates at different sampling heights. The droplet deposition rate at a sampling height of 2 m shows a significant difference compared to the rates at 1 m and 1.5 m sampling heights. However, there is no significant difference in the droplet deposition rates between the 1 m and 1.5 m sampling heights.

**Figure 14:**
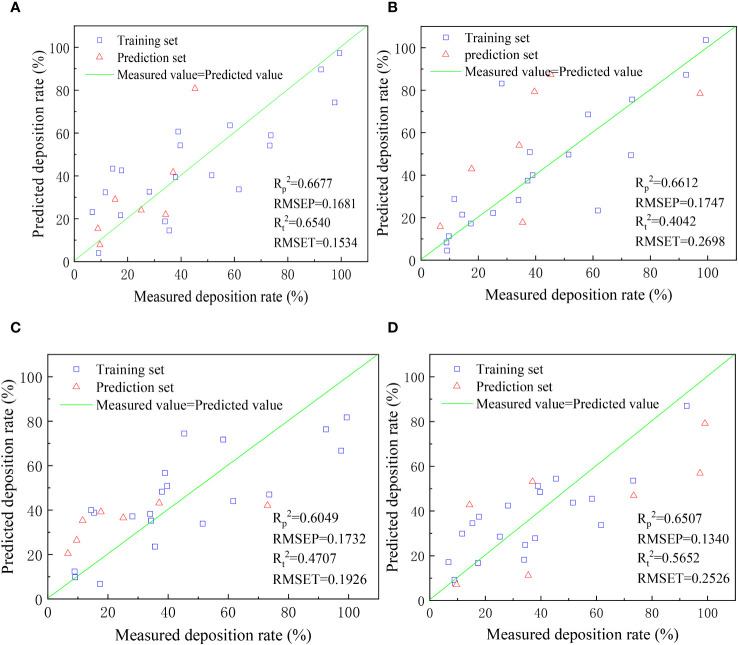
Scatter plot of measured and predicted droplet deposition rates (**(A)** REGRESS, **(B)** BP Neural Network, **(C)** ELM, **(D)** RBFNN).

In order to analyze the relationship between unmanned aerial vehicle (UAV) flight height, droplet size, sampling height, and the deposition rate of droplets on target trees, four different machine learning methods were employed. These methods include REGRESS, BP neural network, ELM, and RBFNN. Prediction models were established using these methods to predict the deposition rate of droplets on target trees. **Figure 14** displays the prediction results obtained from the four machine learning methods.

From the observations in **Figure 14**, it can be seen that none of the four modeling methods achieved satisfactory results in predicting the deposition rate of droplets. The highest coefficient of determination (R^2) obtained among the four methods for both training and prediction sets is 0.6677. The limited success in prediction may be attributed to the weak regularity of the experimental data or the insufficient amount of data available for training and prediction in machine learning models. Moreover, it is noteworthy that the unmanned aerial vehicle flight height, droplet size, and sampling height did not show a significant impact on the deposition rate, which aligns with the conclusion drawn during the data preprocessing stage that these factors do not have a substantial influence on the deposition rate.

### 3.6 Prediction of droplet drift rate

The results of the significance analysis for the DJI T40 crop-spraying UAV experiment between flight height, droplet size, downwind distance, and drift rate are presented in **Table 3**. When examining the impact of different flight heights on the drift rate, a significance analysis was conducted considering three flight heights. According to **Table 3**, it can be inferred that there is no significant relationship between the flight height and the drift rate. However, it is observed that the drift rate tends to increase with higher flight heights. Overall, these results suggest that flight height does not have a statistically significant effect on the drift rate for the DJI T40 crop-spraying UAV experiment. Nevertheless, it should be noted that there is a general trend of increased drift rate with higher flight heights.

**Table 3:** Droplet deposition rates at different flight altitudes, droplet sizes, downwind distances.

		Deposition rate
Flight altitude/m	2	0.043 ± 0.024^a^
	3	0.047 ± 0.030^a^
	4	0.051 ± 0.028^a^
Droplet size/μm	60	0.063 ± 0.029^a^
	100	0.048 ± 0.021^b^
	140	0.030 ± 0.014^c^
Downwind distance/m	3	0.074 ± 0.024^a^
	5	0.054 ± 0.018^b^
	10	0.037 ± 0.013^c^
	15	0.022 ± 0.006^d^

The drift rate data in the table is the mean ± standard deviation; The same small letters indicate that there is no significant difference in droplet drift rate under different downwind distances, and the significance level setting p=0.05.

In the analysis of the effects of different droplet sizes on drift rate, a significance analysis was performed considering three droplet sizes. According to **Table 3**, it can be observed that there is a significant relationship between droplet size and drift rate. Specifically, smaller droplets lead to higher drift rates. This observation aligns with the actual observations, indicating that smaller droplets are more prone to drifting during the DJI T40 crop-spraying UAV experiment.

In the analysis of the effects of different downwind distances on drift rate, a significance analysis was conducted considering four downwind distances. According to **Table 3**, it can be observed that there is a significant relationship between downwind distance and drift rate. The drift rate decreases as the downwind distance increases. This finding aligns with the actual drift pattern of the droplets, indicating that the further the downwind distance, the lower the potential for drift during the DJI T40 crop-spraying UAV experiment.

**Figure 15:**
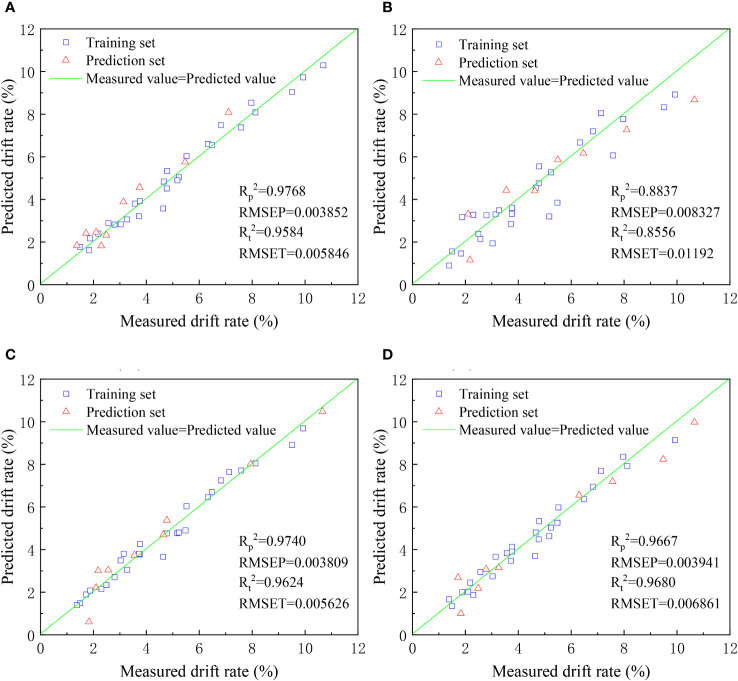
Scatter plot of measured and predicted droplet drift rate (**(A)** REGRESS, **(B)** BP Neural Network, **(C)** ELM, **(D)** RBFNN).

In order to analyze the relationship between flight height, droplet size, downwind distance, and drift rate in plant protection UAV spraying, four machine learning methods were employed: REGRESS, BP neural network, ELM, and RBFNN. These methods were used to establish predictive models for the drift rate of droplets in plant protection UAV spraying. The predictive results of the four machine learning methods are displayed in **Figure 15**.

From **Figure 15**, it can be observed that all four selected modeling methods have good simulation performance in predicting the volume median diameter distribution of droplets. The R_t_^2^ and R_p_^2^ values for the training and prediction sets are all above 0.85. However, the BP neural network model shows relatively poorer performance, with lower values of coefficient of determination (R_t_^2^) and root mean square error compared to the other models. The ELM, REGRESS, and RBFNN models exhibit better and more similar modeling results, with R_t_^2^ and R_p_^2^ values above 0.95 for both the training and prediction sets. These three modeling methods can be effectively applied in predicting droplet drift rates. Among them, ELM demonstrates the smallest root mean square error, making it a preferred choice for predicting droplet drift rates.

## 4 Conclusion

In this study, based on grid atomized droplet technology and machine learning technology, a spraying method combining grid atomization and plant protection UAV is proposed, and the impact test of UAV spraying and grid is carried out to study the principle of grid atomized droplet and the influence of different operating parameters on droplet particle size, deposition and drift, and the spraying model is constructed with machine learning technology to predict the spraying effect of this system. The main research results and conclusions are as follows:

(1) Field experiments were conducted utilizing the DJI T40 plant protection UAV to investigate the deposition rate and downwind drift of droplets on three different types of citrus trees: dwarfed, hedgerow, and densely planted. The experiments were carried out under various conditions, including three droplet sizes (coarse, medium, and fine) and three UAV flight heights (2, 3, and 4 m), both with and without the presence of a grid. The findings yielded from the experimental analysis indicate notable observations. Firstly, the deposition rate of droplets on dwarfed and hedgerow citrus trees was observed to be considerably higher in comparison to traditional densely planted citrus trees. This observation highlights the significant influence of tree type and arrangement on droplet deposition. Secondly, the inclusion of a grid resulted in a slightly reduced deposition rate of droplets on citrus trees as opposed to the absence of a grid. However, this disparity was not deemed statistically significant. Furthermore, when considering the grid condition, there was minimal discrepancy in the deposition rate of droplets on citrus trees between the coarse and fine droplet sizes. This suggests that the use of a grid contributes to a consistent deposition rate irrespective of droplet size. Additionally, it was observed that when employing a coarse droplet size, the drift rate of droplets was lower compared to utilizing a fine droplet size. This finding indicates that opting for a coarser droplet size effectively mitigates drift during pesticide spraying operations. In light of these findings, it can be concluded that deploying a grid on citrus trees along with the utilization of a coarse droplet size facilitates the sustenance of a high deposition rate of droplets on citrus trees while concurrently reducing drift. As a result, there is an improvement in the overall efficiency of pesticide utilization in citrus tree spraying operations. Fixed-height grids have improved droplet coverage and deposition density for both hedgerow and densely planted fruit trees, while dwarf fruit trees are farther away from the grid than other fruit trees, so they have the opposite effect on dwarf fruit trees, and adjustable-height grids will be considered in the subsequent study, while how to arrange the grids more conveniently and solve the problem of cost are also issues that need to be considered when they are put into practical use in the future.
(2) In order to predict the droplet size, deposition rate, and downwind drift of droplets following the impact with the grid, machine learning techniques were employed. Appropriate machine learning methods were carefully selected for prediction and validation, enabling the analysis of the influence of various operational parameters on droplet size, deposition, and drift subsequent to grid collision. The experimental findings indicate that the horizontal distance from the nozzle exerts the greatest impact on the volume median diameter of droplets, followed by the vertical distance from the nozzle. On the other hand, the grid aperture has the least influence on droplet size. Concerning droplet deposition rate, the sampling point height emerges as the most influential factor, whereas UAV flight height and droplet size exhibit negligible effects. In terms of droplet drift rate, the downwind distance is found to have the greatest impact, followed by droplet size, while UAV flight height exerts the least influence. Among the four machine learning methods assessed, the BP neural network and ELM (Extreme Learning Machine) demonstrate favorable performance in predicting droplet size. However, the BP neural network exhibits suboptimal performance in predicting droplet drift rate. On the other hand, ELM, REGRESS, and RBFNN (Radial Basis Function Neural Network) display similar performance characteristics. Therefore, ELM can be given priority when predicting both droplet size and drift rate. To summarize, the utilization of machine learning techniques enables effective prediction of droplet size, deposition rate, and downwind drift after interaction with the grid. The experimental results highlight the varying influences of different operational parameters on these droplet characteristics. Additionally, the evaluation of various machine learning methods identifies ELM as a preferential choice for accurate predictions of droplet size and drift rate.

## Data availability statement

The raw data supporting the conclusions of this article will be made available by the authors, without undue reservation.

## Author contributions

XX: Writing – review & editing, Conceptualization, Data curation, Formal analysis, Methodology, Project administration, Supervision, Writing – original draft. YT: Conceptualization, Data curation, Formal analysis, Investigation, Methodology, Writing – original draft, Writing – review & editing. ZY: Conceptualization, Data curation, Formal analysis, Investigation, Methodology, Writing – original draft, Writing – review & editing. ZL: Funding acquisition, Supervision, Writing – review & editing. SL: Conceptualization, Supervision, Validation, Writing – review & editing. SS: Conceptualization, Supervision, Validation, Writing – review & editing. DS: Conceptualization, Supervision, Validation, Writing – review & editing.
